# Pulmonary function impairment predicted poor prognosis of patients with hepatocellular carcinoma after hepatectomy

**DOI:** 10.18632/oncotarget.20850

**Published:** 2017-09-12

**Authors:** Yanhua Zhao, Shusheng Leng, Dongdong Li, Shu Feng, Zhonghao Wang, Chuanmin Tao

**Affiliations:** ^1^ Department of Laboratory Medicine/Clinical Research Center of Laboratory Medicine, West China Hospital of Sichuan University, Chengdu 610041, China; ^2^ General Surgery Department, Affiliated Hospital/Clinical Medical College of Chengdu University, Chengdu 610081, China

**Keywords:** hepatocellular carcinoma, pulmonary function, hypoxia, prognosis

## Abstract

Tumor hypoxia can influence the progression and metastasis of various cancers, including hepatocellular carcinoma (HCC). Clinical studies have indicated that hyperbaric oxygen may improve the prognosis and reduce complications in HCC patients; however, whether pulmonary function can influence the prognosis of HCC remains unknown. In this study, we found that pulmonary function was associated with clinicopathological features, including smoking, liver cirrhosis, tumor size Edmondson-Steiner grade, total operative blood loss and perioperative blood transfusion. Through Cox proportional hazard regression analysis, smoking, tumor number, tumor size, liver cirrhosis, total operative blood loss and pulmonary function were independent risk factors for overall survival (OS) and disease-free survival (DFS). In addition, poor pulmonary function was independently associated with shorter survival and increased HCC recurrence in patients. Notably, we also found that HCC with liver cirrhosis predicted worse prognosis. In summary, our study found pulmonary function could influence HCC progression. Improve pulmonary function may enhance the OS and DFS of patients with HCC.

## INTRODUCTION

Hepatocellular carcinoma (HCC) is the fifth most commonly diagnosed cancer in males and the ninth in females, with an estimated 782 500 new cancer cases and 745 500 deaths worldwide occurring in 2012. It is the second leading cause of cancer-related death in men and the sixth in women [1]. Especially, China makes up about 50% of the total number of cases and deaths alone. There are an estimated 343700 patients died of HCC in 2015 [2]. Etiology study shows chronic hepatitis B virus (HBV) infection is the major causes of HCC in Asia and the other factors including hepatitis C virus (HCV) infection, alcohol abuse, smoking, autoimmune hepatitis, and several metabolic diseases are considered as the minority causes [1]. Despite rapid progression advanced treatments including surgical techniques and other therapies, the prognosis of HCC patients is still poor due to high frequency of metastasis and recurrence [3, 4].

Hypoxia is well-recognized solid tumor characteristics, which confers tumor cell aggressive behaviors, including promoting proliferation, invasion and metastasis [5, 6]. Evidences support that acute or chronic hypoxia of tumors predicts a negative prognosis of cancer patients owing to drug resistance and distant metastasis [7]. The microenvironment of stiff and hypoxia promotes the development of breast cancer stem-like cells (BCSC) through modulation of the integrin-linked kinas ILK [8]. The BCSC phenotype was induced by hypoxia in an HIF- and ALKBH5-dependent manner [9]. In addition, tumor hypoxia hypermethylates the promoters of tumor suppressor genes by reducing TET oxygen-dependent ten-eleven translocation (TET) enzymes activity. With DNA hypermethylation, the tumor suppressor genes are transcriptionally repressed, conferring growth advantages to tumor cells [10]. On hypoxic gradients, cancer cells not only migrate faster but also extend over a longer distance [11]. Moreover, tumor metabolism switches toward glycolysis, antagonizes apoptosis and autophagy, dampens oxidative stress, maintaining tumor cell proliferation in the face of severe hypoxia [12].

HCC is one of the most hypoxic cancer with its multiple nodules scavenging a substantial amount of oxygen [13]. A growing body of literatures report that HIF1α is significantly elevated and associates with clinical outcome in HCC [14–16]. Hypoxia-targeted therapeutic strategies for HCC may be under a promising value. Improvement of hepatoma hypoxia inhibits cancer growth and migration [17]. The acute hyperbaric oxygen therapy (HBOT) after hepatectomy improves the progression of HCC [18].

Albeit many studies focus on intratumoral hypoxia, seldom research pays attention to tumor systemic hypoxia. Pulmonary function impairment is a critical factor for systemic hypoxia. However, whether pulmonary function influence the prognosis of HCC remains unknown. The present study was undertaken to investigate the impact of pulmonary function in HCC patients who had undergone hepatectomy.

## RESULTS

### Clinicopathologic features of 115 patients with HCC

In order to analyze clinicopathologic features of 115 HCC patients, the patients were divided into three groups according to different pulmonary function. There were 54 patients in normal pulmonary function group, 34 patients in pulmonary function slightly impaired group, and 27 patients in pulmonary function moderately impaired group. The diagnosis of HCC was confirmed histopathologically. The clinicopathologic parameters of these patients are summarized in Table 1. Among three groups, there were substantial differences in smoking (*P*=0.007), liver cirrhosis (*P*<0.001), tumor size (*P*=0.006) and Edmondson-Steiner grade (*P*<0.001).

**Table 1:** Clinicopathologic features of 115 HCC patients in different pulmonary function groups

Clinicopathologic variable	No.	Pulmonary function			*P* value
		Normal	Slightly impaired	Moderately impaired	
**Gender**					
Female	30	18	5	7	0.153
Male	85	36	29	20	
**Smoking**					
No	50	30	15	5	**0.007**
Yes	65	24	19	22	
**HBsAg**					
Positive	90	40	27	23	0.511
Negative	25	14	7	4	
**Liver cirrhosis**					
Presence	70	39	28	13	**<0.001**
Absence	45	15	6	22	
**AFP**					
≥400μg/L	69	32	17	20	0.161
< 400μg/L	56	22	17	7	
**Tumor number**					
Solitary	74	34	18	22	0.066
Multiple (≥2)	41	20	16	5	
**Tumor size**					
≤5 cm	42	28	8	6	**0.006**
>5 cm	73	26	26	21	
**Vascular invasion**					
Presence	39	15	14	10	0.261
Absence	76	39	26	11	
**Capsular formation**					
Presence	35	20	10	5	0.023
Absence	80	34	24	22	
**Child-Pugh**					
A	75	35	20	20	0.461
B	40	19	14	7	
**Edmondson-Steiner grade**					
Low grade (I and II)	69	22	28	19	**<0.001**
High grade (III and IV)	46	32	6	8	
**BCLC Stage**					
0/A	53	25	15	13	0.951
B/C	62	29	19	14	
**Clip scores**					
0-2	76	38	19	19	0.326
3-4	39	16	15	8	
**ALBI grade**					
1	53	29	14	10	0.548
2	41	17	12	12	
3	21	8	8	5	

### Intraoperative and postoperative data of 115 HCC patients

The operative and postoperative data in different pulmonary function groups are summarized in Table 2. The total operative blood loss for the impaired pulmonary function groups was greater than the normal pulmonary function group (*P*=0.032). Compared with the normal pulmonary function group, there were more patients in the impaired pulmonary function groups who required perioperative blood transfusion (*P*=0.048). However, there were no substantial differences in time of hepatic inflow exclusion, time for operation, surgical margin, hospital stay and major complications among three groups.

**Table 2:** Operative and postoperative data in different pulmonary function groups

variable	Pulmonary function			*P* value
	Normal (n=54)	Slightly impaired(n=34)	Moderately impaired(n=27)	
**Time of hepatic inflow exclusion (min)**	19.6 ± 12.8	18.2 ± 11.9	20.3 ± 10.8	0.191
**Time for operation (min)**	138±47	129 ±50	145 ± 55	0.547
**Total operative blood loss (ml)**				
<1000	40	22	12	**0.032**
>1000	14	12	15	
**Perioperative blood transfusion**				
Without	39	20	12	**0.048**
With	15	14	15	
**Surgical margin**				
>1.0 cm	45	24	21	0.368
≤1.0 cm	9	10	6	
**Hospital stay, days**	13.2 ± 4.5	14.4 ± 5.6	17.1 ± 6.2	0.428
**Major complications (%)**	9 (17%)	8 (24%)	11 (41%)	0.058
Ascites	3	3	2	0.837
Wound infection	2	1	1	0.979
Pleural effusion	1	2	4	0.071
Liver failure	1	0	2	0.176
Biliary fistula	1	2	1	0.602
Bleeding	1	0	1	0.545

### Pulmonary function impairment was an independent predictor for the poor prognosis of HCC

For further investigate the clinical impact of pulmonary function impaired to HCC patients, pulmonary function impaired was checked whether it was risk factors for overall survival (OS) and disease-free survival (DFS). By Cox proportional hazard regression analysis, smoking (Hazard ratios (HR): 1.952, *P*=0.020), tumor number (HR: 2.214, *P*=0.035), tumor size (HR: 2.625, *P*=0.017), liver cirrhosis (HR: 2.648, *P*=0.002), Edmondson-Steiner grade (HR: 1.842, *P*=0.010), pulmonary function (HR: 1.788, *P*<0.001) and total operative blood (HR: 2.874, *P*=0.042) had poor OS rates than those without these variables on univariate analysis. However, on multivariate analysis, smoking (HR: 2.115, *P*=0.032), tumor size (HR: 2.765, *P* = 0.012), liver cirrhosis (HR: 1.928, *P*=0.018), pulmonary function (HR: 1.721, *P* = 0.001) and total operative blood loss (HR: 2.576, *P*=0.042) were recognized as independent predictors of postoperative OS (Table 3).

**Table 3:** Univariable and multivariable analysis of overall survival(OS) and pulmonary function by Cox proportional hazards regression model

Variables	Univariable Analysis		Multivariable Analysis	
	HR (95% CI)	*P* Value	HR (95% CI)	*P* Value
**Gender** (Male *vs.* Female)	0.760(0.430-1.916)	0.350		NA
**Smoking** (No *vs.* Yes)	1.952(1.590-5.280)	**0.020**	2.115(1.203-6.182)	**0.032**
**HBsAg** (Positive *vs.* Negative)	0.347(0.216-1.230)	0.410		NA
**AFP** (≥400μg/L *vs.* < 400μg/L)	4.162(0.658-8.105)	0.557		NA
**Tumor number** (Solitary *vs.* Multiple)	2.214(1.965-5.232)	**0.035**	2.314(0.776-5.247)	0.710
**Tumor size** (≤5 cm *vs.*>5 cm)	2.625(1.870- 5.395)	**0.017**	2.765(1.540-5.327)	**0.012**
**Vascular invasion** (Absent *vs.* Present)	3.070(0.605-6.832	0.112		NA
**Capsular formation** (Present *vs.* Absent)	2.455(0.730-3.460)	0. 320		NA
**Liver cirrhosis** (Absent *vs.* Present)	2.648(1.581-7.244)	**0.002**	1.928(1.365-4.328)	**0 .018**
**Child-Pugh** (A *vs.* B)	1.756(0.306-3.780)	0.805		NA
**Edmondson-Steiner grade** (I-II *vs.* III-IV)	1.842(1.234-3.183)	**0.010**	1.988 (0.793-3.949)	0.230
**BCLC Stage** (0/A *vs.* B/C*.*)	3.742(0.560-6.641)	0.170		NA
**Clip scores** (0-2 *vs.* 3-4)	2.441 (0.788-2.032)	0.562		NA
**ALBI grade** (1 *vs.*2 *vs* 3)	1.213 (0.551-2.669)	0.632		NA
**Pulmonary Function** (Normal ***vs.*** Slightly ***vs.*** Moderately)	1.788(1.324-2.416)	**<0.001**	1.721 (1.264 - 2.344)	**0.001**
**Total operative blood loss** (ml) (≤1000 ***vs.***>1000)	2.874(1.156-7.145)	**0.025**	2.576(1.263- 5.054)	**0.042**
**Perioperative blood transfusion** (Absent ***vs.*** Present)	1.788(0.492-6.420)	0.149		NA
**Surgical margin** (≥1cm *vs.* < 1cm)	3.275(0.768-9.287)	0.097		NA

Further study showed that smoking (HR: 2.357, *P*=0.013), tumor number (HR: 1.657, *P*=0.019), tumor size (HR: 2.153, *P*=0.008), liver cirrhosis (HR: 2.162, *P*=0.037), Edmondson-Steiner grade (HR: 1.650, *P*=0.016), pulmonary function (HR: 2.537, *P*<0.011) and total operative blood loss (HR:1.323, *P*=0.031) had poor DFS on univariate survival analysis (Table 4). Furthermore, smoking (HR: 2.160, *P*=0.035), the tumor size (HR: 1.915, *P*=0.043), liver cirrhosis (HR: 2.416, *P*=0.026), pulmonary function (HR: 2.839, *P*=0.010) and total operative blood loss (HR: 2.403, *P*=0.039) were independent predictors of postoperative DFS on multivariate analysis (Table 4). That demonstrated pulmonary function impairment was a risk factor for predicting the poor prognosis of HCC after operation.

**Table 4:** Univariable and multivariable analysis of disease-free-survival (DFS) and pulmonary function by Cox proportional hazards regression model

Variables	Univariable Analysis		Multivariable Analysis	
	HR (95% CI)	*P* Value	HR (95% CI)	*P* Value
**Gender** (Male *vs.* Female)	1.953(0.571-4.035)	0.252		NA
**Smoking** (No *vs.* Yes)	2.357(1.264-8.660)	**0.013**	2.160(1.154-7.497)	**0.035**
**HBsAg** (Positive *vs.* Negative)	0.825(0.409- 1.862)	0.265		NA
**AFP** (≥400μg/L *vs.* < 400μg/L)	0.749(0.517- 2.229)	0.081		NA
**Tumor number** (Solitary *vs.* Multiple)	1.657(1.164-5.640)	**0.019**	1.873(0.650-6.246)	0.526
**Tumor size** (≤5 cm *vs.*>5 cm)	2.153 (1.773-3.069)	**0.008**	1.915(1.252- 3.103)	**0.043**
**Capsular formation** (Absent *vs.* Present)	1.244 (0.733-3.039)	0.246		NA
**Liver cirrhosis** (Present *vs.* Absent)	2.162 (1.245-3.276)	**0.037**	2.416(1.228-3.712)	**0.026**
**Vascular invasion** (Absent *vs.* Present)	2.129 (0.328-4.314)	0.158		NA
**Child-Pugh** (A *vs.* B)	1.781(0.516-3.481)	0.581		NA
**Edmondson-Steiner grade** (I-II *vs.* III-IV)	1.650 (1.520-2.855)	**0.016**	1.368 (0.745-3.689)	0.547
**BCLC Stage** (0/A *vs.* B/C*.*)	1.518(0.522- 2.928)	0.354		NA
Clip scores (0-2 *vs.* 3-4)	1.815(0.898-3.669)	0.130		NA
**ALBI grade** (1 *vs.*2 *vs* 3)	1.929(0.824-4.515)	0.441		NA
**Pulmonary Function (**Normal *vs.* Slightly *vs.* Moderately)	2.537(1.924–8.364)	**0.011**	2.839(1.884-8.477)	**0.010**
**Total operative blood loss** (ml) (≤1000 ***vs.***>1000)	1.323(1.075-2.232)	**0.031**	2.403(1.104-17.560)	**0.039**
**Perioperative blood transfusion** (Absent ***vs.*** Present)	1.885(0.957- 3.715)	0.272		NA
**Surgical margin** (≥1cm *vs.* < 1cm)	3.441(0.330-8.904)	0.632		NA

### Overall and disease-free survival curves of HCC patients with different pulmonary function

**Figure 1:**
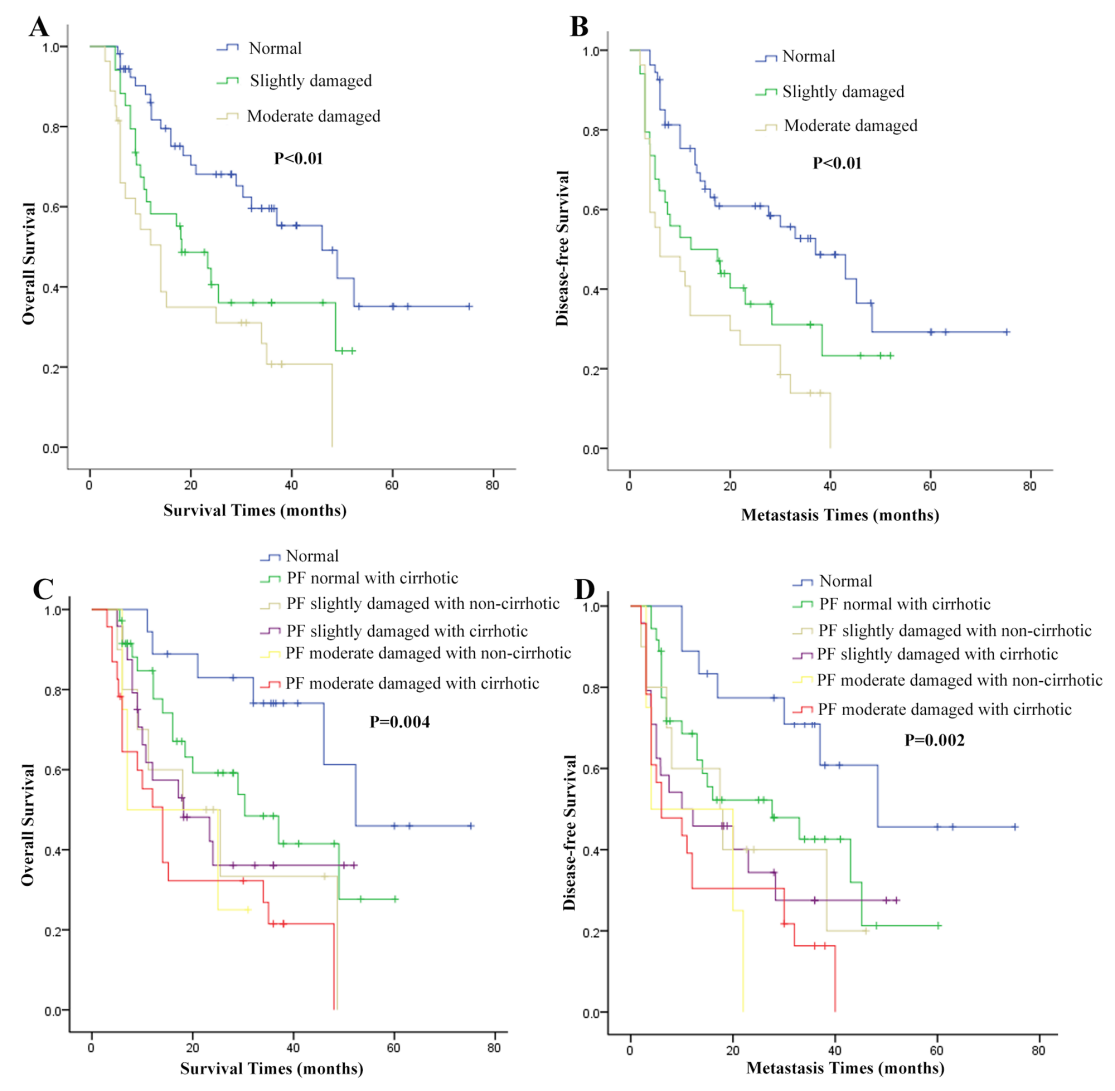
Overall survival (OS) and disease-free survival (DFS) curves in hepatocellular carcinoma (HCC) patients with different pulmonary function (PF) and combined cirrhotic or not **(A and B)** show that HCC patients with pulmonary function impaired had lower OS and DFS time than those with normal pulmonary function (*P*<0.01 and *P*<0.01). **(C and D)** show that HCC patients with impaired pulmonary function and cirrhosis had significantly lower OS and DFS time than other groups (*P*=0.004 and *P*=0.002).

In order to study pulmonary function on the prognosis of HCC patients after hepatectomy, the 1-, 3-, 5-year OS rates and DFS rates among three groups were analyzed. In our study, the proportion of tumor recurrence rate was 55.7%(64/115) and DFS rate was 44.3%(51/115) in 115 HCC patients. Then, Kaplan-Maier analysis was exploited to investigate the patient OS and DFS rates. The survival curves showed that HCC patients with pulmonary function impaired had lower OS and DFS time than those with normal pulmonary function (Figure 1A, 1B). For normal pulmonary function patients, the 1-, 3-, and 5-year OS rates were 88%, 60%, 37%, respectively. For pulmonary function slightly impaired patients, the 1-, 3-, and 5-year OS rates were 61%, 35%, 17%, respectively. For pulmonary function moderately impaired patients, the 1-, 3-, and 5-year OS rates were 55%, 22%, 0%, respectively (Table 5). The OS and DFS were greater in patients with normal pulmonary function (*P*<0.01).

**Table 5:** 1-, 3-, and 5-year overall survival rates in different groups

Groups	Overall Survival rate		
	1-year	3-year	5-year
Normal pulmonary function	88%	60%	37%
Pulmonary function slightly impaired	61%	35%	17%
Pulmonary function moderately impaired	55%	22%	0%
Normal pulmonary function with cirrhosis	60%	42%	28%
Pulmonary function slightly damaged with cirrhosis	42%	35%	0%
Pulmonary function moderately damaged with cirrhosis	32%	22%	0%

### Liver cirrhosis may make HCC patients with pulmonary function impaired worse prognosis

Hypoxia is the characteristic microenvironment of chronic hepatitis [19]. HIFs activation contributes to liver fibrosis development [20]. We explored whether the incremental cirrhotic condition accelerates poor prognosis or not. The results of clinicopathologic parameters showed that liver cirrhosis had potential differences among three groups. For further study, we divided the three groups into six groups according to the presence of liver cirrhosis or not (Figure 1C, 1D). Noteworthy, subgroup analysis showed that moderately pulmonary function with cirrhotic had significantly lower OS and DFS time than normal pulmonary function without cirrhotic group (*P*=0.004 and *P*=0.002). Furthermore, the 1-, 3-, and 5-year OS rates in patients with damaged pulmonary function and cirrhosis were lower than those in normal pulmonary function group (Table 5). These results demonstrated that pulmonary function may affect the prognosis of HCC patients after hepatic resection. Liver cirrhosis may accelerate this process. Hypoxia may influence the survival of long-term survival.

## DISCUSSION

In this study, we found that pulmonary function impaired was associated with poor clinicopathological features including smoking, liver cirrhosis, tumor size and Edmondson-Steiner grade. Through Cox proportional hazard regression analysis, we also discovered that pulmonary function and liver cirrhosis were independent risk factors for OS and DFS. In addition, pulmonary function impairment predicted lower OS and DFS time. The underlying reason may be due to the low oxygen level in patients with pulmonary function impairment. Previous evidences support that hypoxic microenvironment stimulates cancer cells proliferation, angiogenesis, treatment resistance and metastasis [5, 6]. Hypoxia protects cancer cells from chemotherapy induced apoptosis through both HIF-1α-mediated and HIF-1α-independent in HCC [21, 22]. HCC is a hyper vascular cancer, but the cancer vessels are aberrant both in structure and function [23, 24]. Therefore, the prognosis of HCC is poor due to cancer hypoxia [21, 25].

Oxygen performs an essential function in the living body, which transports around the body by the vasculature from lung [26]. Pulmonary function testing can reflect the physiological state of lung and evaluate the pulmonary function reserve [27]. FEV1/FVC as gold classification was used to evaluate pulmonary function. It is reported that FEV1/FVC was associated with poor prognosis of malignant cancer, such as lung cancer and colorectal cancer [28, 29]. Decreased FEV1/FVC can reflect the systemic hypoxic state of the patients. Our results were parallel with previous findings that the decreased FEV1/FVC had the negative impact on cancer prognosis. It was that impaired pulmonary function might lead to a lower oxygen content in the focal region, which may aggravate intratumoral hypoxia.

Moreover, patients with pulmonary function impaired and cirrhosis had worse prognosis than those without cirrhosis. The potential explanation was that liver cirrhosis might aggravate tumor hypoxia. Hypoxia is the characteristic microenvironment of chronic hepatitis [19, 30]. Liver cirrhosis is characterized by the excessive deposition of extracellular matrix (ECM) proteins such as type I collagen in the liver parenchyma. This is the manifestation of a wound healing response to persistent or repeated injury [31]. With chronic liver disease, abnormal angiogenesis aggravates the hypoxia of liver. Of note the pattern of fibrosis varies according to the underlying disease [30]. In hypoxic microenvironment condition, HIF-1s is a vital regulator of the response of oxygen deprivation and plays critical roles in the adaptation of cancer cells [5]. HIF-1α expression activates hepatic stellate cells (HSCs) and fibroblasts, which differentiates towards to myofibroblasts. The latter proliferates and migrates to injured areas where they secrete ECM [32–34]. This procession in turn makes it worse for hypoxia. So, in our results showed that, compared with impaired pulmonary function groups, impaired pulmonary function with cirrhosis groups had significantly lower OS and DFS time.

Taken together, our results showed that pulmonary function impaired might predict poor prognosis of HCC patients after hepatectomy. Improving the pulmonary function may increase the postoperative survival rate of HCC. However, there are some limitations in our study, and further studies are needed to clarify how to improve the pulmonary function and whether the HCC patients need long-term oxygen therapy.

## MATERIALS AND METHODS

### Patients

From 2011 to 2015, a total of 115 HCC patients underwent liver resection from the Affiliated Hospital of Chengdu University and West China Hospital of Sichuan University were recruited into this study. The patients died during perioperative period were excluded. All patients in our study did not receive any neoadjuvant chemotherapy or local ablation before hepatectomy. Pulmonary function was evaluated according to gold classification. FEV1/FVC ratio (also called Tiffeneau-Pinelli index), the main parameter used for the classification of the patients, represents the proportion of a patient’s vital capacity that they are able to expire in the first second of forced expiration to the full vital capacity. Patients were classified as follow. Normal: FEV1/FVC%≥70; Slight: FEV1/FVC%<70, FEV1%≥80; Moderate: FEV1/FVC%<70, 50≤FEV1%<80; Severe: FEV1/FVC%<70, FEV1%<50 [35].

Of these 115 patients, 54 patients with normal pulmonary function were classified as the normal group, and 34 patients with pulmonary function slightly impaired were classified as the slightly impaired group, whereas 27 with pulmonary function moderately impaired were classified as the moderately impaired group. There were no patients with pulmonary function severely impaired and very severely damaged included in this study. Prior informed consent was obtained from all patients and the study protocol was approved by the Ethics Committee of Affiliated Hospital of Chengdu University.

### Follow-up and prognostic study

The follow-up data were regularly collected by the same experienced team. For all patients, the latest follow-up was terminated on March, 2017. OS was defined as the time from hepatectomy to death or to the date of the last follow-up. DFS was defined as the time from hepatic resection to the date when recurrence or metastasis was detected. Liver function tests, alpha fetoprotein (AFP), ultrasonography and chest radiography were performed every 3 months. If recurrence or metastasis was suspected, computed tomography (CT) scan and/or magnetic resonance imaging (MRI) were performed. Once tumor relapsed, repeat hepatectomy, radiofrequency ablation, or transcatheter arterial chemoembolization (TACE) were used to treat these patients, depending on the number and position of the recurrent tumors, the general condition of the patient, and the liver function.

### Statistical analysis

Statistical analysis was performed by SPSS 21.0 software. Categorical variables were compared using the Pearson χ^2^. All continuous data were presented as mean ± SD and were compared by the Student t test. OS and DFS curves were obtained using the Kaplan-Meier method, and differences in survival were evaluated using the log-rank test. Univariate and multivariate analysis were analyzed with Cox proportional hazards regression model to verify the independent risk factors. All potential variables were included in Cox Univariable analysis. Then the variables with significant *P* value (*P* < 0.05) were included in Cox multivariable analysis. Using STATA software to check whether the assumed hazard ratio comply with the assumption of proportionality beforehand. 2-side *P* value< 0.05 was considered to be statistically significant.
